# Isolated oculomotor nerve palsy as a paraneoplastic manifestation of gastric diffuse large B-cell lymphoma: A case report

**DOI:** 10.3892/ol.2014.2454

**Published:** 2014-08-19

**Authors:** SHANG-YIH YAN, YI-JEN PENG, CHUN-SHU LIN, GIIA-SHEUN PENG, PING-YING CHANG

**Affiliations:** 1 Department of Neurology, Tri-Service General Hospital, National Defense Medical Center, Taipei 114, Taiwan, R.O.C.; 2 Department of Pathology, Tri-Service General Hospital, National Defense Medical Center, Taipei 114, Taiwan, R.O.C.; 3 Department of Radiation Oncology, Tri-Service General Hospital, National Defense Medical Center, Taipei 114, Taiwan, R.O.C.; 4 Division of Hematology/Oncology, Department of Internal Medicine, Tri-Service General Hospital, National Defense Medical Center, Taipei 114, Taiwan, R.O.C.

**Keywords:** oculomotor nerve palsy, paraneoplastic, diffuse large B-cell lymphoma

## Abstract

Isolated oculomotor nerve palsy (ONP) is rare. The major causes are aneurysm of the posterior communicating artery, microvascular ischemia, neoplasm, inflammation and trauma. The present study reports the case of a 72-year-old female with left isolated pupil-sparing ONP and severe anemia as the initial manifestations of gastric diffuse large B-cell lymphoma (DLBCL). Systemic chemotherapy without any central nervous system (CNS)-directed treatment led to a complete resolution of the ONP, suggesting that it was most likely to be a paraneoplastic phenomenon. If CNS involvement cannot be demonstrated by brain magnetic resonance imaging and cerebrospinal fluid assessment, the present case suggests that it may be acceptable to omit CNS-directed therapy in such cases of ONP, since it may be paraneoplastic in nature and may resolve following successful treatment of the underlying malignancy.

## Introduction

The gastrointestinal tract is the most common site for primary extranodal lymphoma which accounts for ≤40% of all cases (1,2). The most common location for GI tract involvement is the stomach (1,2). Gastric diffuse large B-cell lymphoma (DLBCL) is a common subtype. Gastric DLBCL may be treated with surgical resection, chemotherapy and radiotherapy either alone or in combination. Surgical intervention may be reserved for treating complications such as major bleeding, obstruction or perforation (2). Conservative treatment with chemotherapy with or without radiotherapy is the preferred treatment method (3,4). According to previous retrospective studies, rituximab, in combination with chemotherapy, has a higher complete response, event-free survival and overall survival rates compared with conventional chemotherapy (5,6). Lymphomas can directly, by neoplastic infiltration, or indirectly, by a paraneoplastic or infectious cause, involve the peripheral and central nerves (7,8). Multiple cranial nerve involvement is not uncommon in lymphoma, but isolated neuropathy is rare. The facial nerve is the most vulnerable to leptomeningeal disease (9,10). Isolated oculomotor nerve palsy (ONP) from lymphoma is also extremely rare (11,12). The diagnosis and management of ONP are directed by the age of the patient and by the degree to which the third nerve major functions (pupillomotor, oculomotor) have been affected. The outcome of ONP is related to its cause (13). The involvement of the central nervous system (CNS) at the time of diagnosis in DLBCL is uncommon and the majority of the events occur during relapse, with an incidence ranging from 2–7% (14–16). High-dose methotrexate with or without CNS-directed therapy is normally used for disease control. The patient prognosis is poor, despite treatment options being available (17,18). The present study reports the case of a patient with gastric DLBCL with isolated pupil-sparing ONP. Written informed consent was obtained from the patient.

## Case report

**Figure 1:**
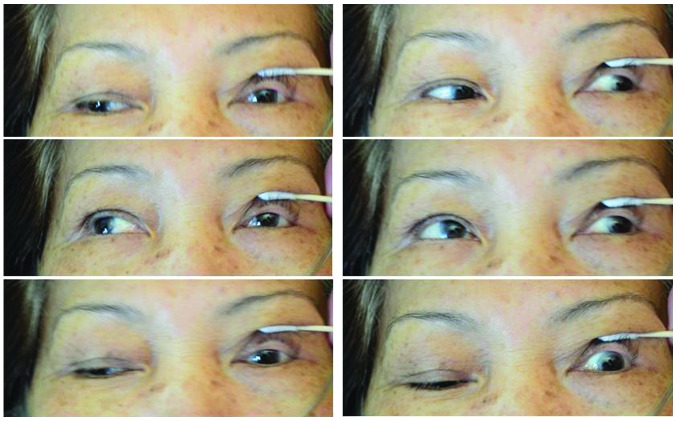
Exotropia, hypotropia, impaired adduction and ptosis of the left eye whilst gazing to the left and right in horizontal, upward and downward directions, respectively.

**Figure 2:**
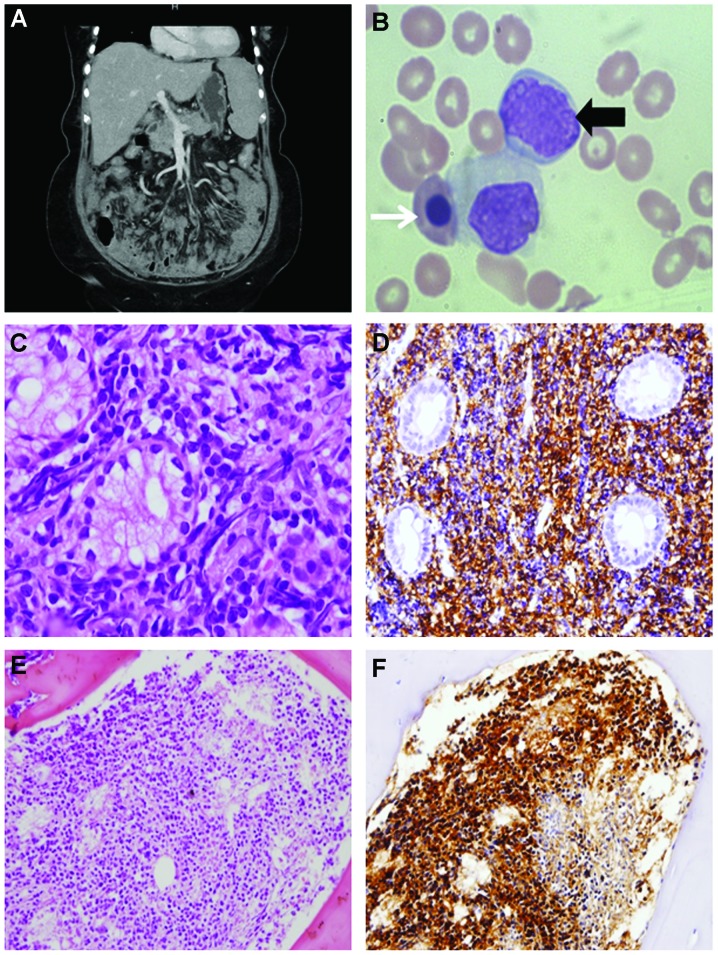
(A) Abdominal computed tomography scan showing diffuse and numerous nodular lesions over the mesentery, para-aortic spaces and bilateral iliac chains. (B) Peripheral blood smear showing a nucleated red blood cell (arrowhead) and myeloblasts (black arrow) with a high nucleus/cytoplasm ratio and conspicuous nucleoli. (C) Medium- to large-sized lymphocytes infiltrating the gastric mucosa [hematoxylin and eosin (HE) stain; magnification, ×1,000] that are (D) immunoreactive for the B lymphocyte marker, cluster of differentiation (CD)20 (magnification, ×400). (E) The intertrabecular marrow space was occupied by the same tumor cells with patches of tumor necrosis (HE; magnification, ×400) and (F) these cells were also immunoreactive for CD20 (×400).

A 72-year-old female with a medical history of hypertension presented with left ptosis and diplopia, easy-onset fatigue and abdominal fullness that had persisted for two weeks. Upon admission, the patient was afebrile with normal vital signs, but appeared lethargic. Physical examination revealed pale conjunctiva, mild upper abdominal tenderness and splenomegaly 2 cm below the left costal margin. There was no overt lymphadenopathy. In a neurological examination, the patient exhibited left ONP with ptosis and external ophthalmoplegia (Fig. 1). There were no pupillary or visual abnormalities. Other cranial nerves, muscle power, sensation, coordination and reflexes were normal. Laboratory studies revealed a white blood cell count of 2,460/μl (normal range, 4,500–11,000/μl), a hemoglobin level of 4.9 g/dl (normal range, 12–16 g/dl), a mean corpuscular volume of 80.7 fl (normal range, 80–100 fl) and a platelet count of 145×10^3^/μl (normal range, 150–400×10^3^/μl), with myeloid precursors and nucleated red blood cells in the peripheral smear (Fig. 2A). The patient also had a serum ferritin level of 1,480 ng/ml (normal range, 20–300 ng/ml), a serum lactic dehydrogenase level of 2,007 U/l (normal range, 115–245 U/l) and occult blood was present in the stool. Gadolinium-enhanced brain magnetic resonance imaging (MRI) displayed no abnormality of the cavernous sinus and no leptomeningeal disease. The cranial nerves were well delineated without abnormal enhancement. A contrast-enhanced computed tomography scan of the abdomen revealed thickening of the wall of the gastric antrum and diffuse intra-abdominal lymphadenopathy (Fig. 2B). Panendoscopy revealed multiple polypoid lesions with ulcers in the gastric antrum and body. These were biopsied and were found to be consistent with DLBCL upon histopathological assessment (Fig. 2C and D). A bone marrow biopsy was performed that revealed lymphoma involvement (Fig. 2E and F), and [^18^F]-fluorodeoxyglucose (FDG) positron emission tomography revealed multiple FDG-avid lesions in the entire skeleton, as well as within the abdomen. The patient was therefore diagnosed with Ann Arbor stage IVB gastric DLBCL with involvement of the bone marrow and left-sided isolated ONP. A lumbar puncture was performed and revealed a negative finding. The patient received systemic chemotherapy with 375 mg/m^2^ rituximab on day 1, 750 mg/m^2^ cyclophosphamide on day 1, 1.4 mg/m^2^ vincristine on day 1 and 100 mg prednisolone on days 1–5, every three weeks. Anthracyclines were not administered during the treatment due to the patient’s poor overall condition and the high risk of toxicity. The symptom of left ONP was resolved completely two weeks after the first cycle of chemotherapy.

## Discussion

Lesions arising anywhere along the course of the oculomotor nerve, including the nucleus, the fascicles in the midbrain tegmentum and the spaces it passes through, including the subarachnoid space, the cavernous sinus and the superior orbital fissure, can lead to palsy of the nerve. The major causes of isolated third nerve palsy include aneurysms of the posterior communicating artery, microvascular ischemia, neoplasm, inflammation and trauma (13). Microvascular ischemia, which is often associated with diabetes mellitus and hypertension, is believed to be the most common cause of isolated pupil-sparing third nerve palsies. However, compression by aneurysms or tumors can also lead to pupil-sparing ONP (19). Neuroimaging, such as brain MRI, is suggested for patients with such presentation.

Sato *et al* (20) reviewed 14 lymphoma cases presenting with ONP, and ten out of 14 were assessed by using brain MRI, which detected eight patients with CNS involvement. The majority of the patients with pupil-sparing ONP exhibited cavernous sinus involvement rather than oculomotor nerve infiltration (16). Brain MRI combined with cerebrospinal fluid (CSF) cytology examination is considered optimal for evaluating the cause of ONP, but may not be diagnostic in every case. In the present patient, brain MRI revealed no significant cranial abnormality and the lumbar puncture examination demonstrated a negative result. Since a diagnosis of lymphoma was made based on gastric and bone marrow biopsies, the patient was able to start systemic chemotherapy and exhibited a good response, including total recovery of the ONP. The fact that the patient recovered without the use of intrathecal or other CNS-directed therapy makes it more likely that the ONP was a paraneoplastic phenomenon and not from lymphomatous involvement of the CNS.

In conclusion, this case demonstrates that isolated ONP associated with gastric DLBCL may represent a paraneoplastic feature of the disease. As such, if brain imaging and CSF examination do not reveal involvement of lymphoma, it may be reasonable to forgo CNS-directed therapy and only treat with appropriate systemic therapy for the underlying disease.
